# A rare case of extramammary Paget disease in a young HIV-positive man

**DOI:** 10.1016/j.jdcr.2023.11.004

**Published:** 2023-11-18

**Authors:** Josephine D’Angelo, Franki Lambert Smith

**Affiliations:** Department of Dermatology, University of Rochester Medical Center, Rochester, New York

**Keywords:** extramammary Paget disease, HIV, Mohs micrographic surgery

## Introduction

Extramammary Paget disease (EMPD) is a rare adenocarcinoma that originates from the skin or skin appendages in apocrine gland–rich areas.^1^, ^2^, ^3^, ^4^, ^5^, ^6^, ^7^, ^8^ Although EMPD is typically limited to the epidermis, it can involve dermis, and if invasive, metastasize to regional lymph nodes and other organs.^2^^,^^5^^,^^6^

EMPD is more common in Caucasian postmenopausal women but also occurs in men. Frequently affected sites include the vulva, followed by the perianal region, scrotum, penis, and axilla.^1^^,^^4^, ^5^, ^6^ EMPD is classified as primary or intraepithelial in most cases; however, it can be secondary and associated with underlying carcinoma or distant tumors.^1^^,^^5^^,^^6^ Associated malignancies include those of the vulva, vagina, cervix and uterus, bladder, ovary, gallbladder, liver, breast, colon, and rectum.^1^^,^^6^

EMPD presents as a slow-growing, erythematous plaque with scale.^1^^,^^2^^,^^6^ It may be asymptomatic or associated with a burning sensation and pruritus.^1^^,^^4^, ^5^, ^6^, ^7^ Establishment of precise boundaries with normal-appearing skin is limited by subclinical extension.^1^^,^^6^

Diagnosis of EMPD is clinical and confirmed by histology. Histopathology reveals large atypical cells with prominent nuclei and mucin-rich cytoplasm in the epidermis.^1^^,^^6^ Immunohistochemistry (IHC) with cytokeratin 7 (CK7) is important for diagnosis.^1^ IHC is useful to distinguish EMPD from other diagnoses such as Bowen disease or amelanotic superficial spreading melanoma and to determine primary vs secondary disease.^6^ A diagnostic IHC panel for EMPD is recommended, including CK7-positive, p63-negative, SOX10-negative, and CEA-positive results.^5^ CK20 and GCDFP-15 can be used to help differentiate primary from secondary EMPD.^5^^,^^6^

EMPD can occur as early as 5 years before an associated internal malignant neoplasm.^5^ Therefore, a diagnosis of EMPD warrants exclusion of underlying malignancy and age-appropriate cancer screening.^1^^,^^5^^,^^6^ Preferred treatment of EMPD includes wide local excision or Mohs micrographic surgery (MMS).^1^^,^^4^, ^5^, ^6^ However, even with surgical treatment, relapses frequently occur because of extensive subclinical disease.^1^^,^^6^ Additional therapies include photodynamic therapy, imiquimod 5% cream, 5-fluorouracil 5% cream, and CO_2_ laser.^1^^,^^4^, ^5^, ^6^ Radiotherapy can be employed as adjuvant treatment after surgery for persistent or recurrent EMPD or when nonsurgical treatment is preferred.^1^^,^^4^^,^^5^ Chemotherapy, targeted therapy, or immune checkpoint inhibitors can be considered for metastatic disease.^5^ For primary EMPD, close long-term clinical follow-up is recommended.^1^^,^^5^^,^^6^

The following case demonstrates a rare presentation of EMPD in a young adult male patient with a history of well-controlled HIV.

## Case report

**Fig 1:**
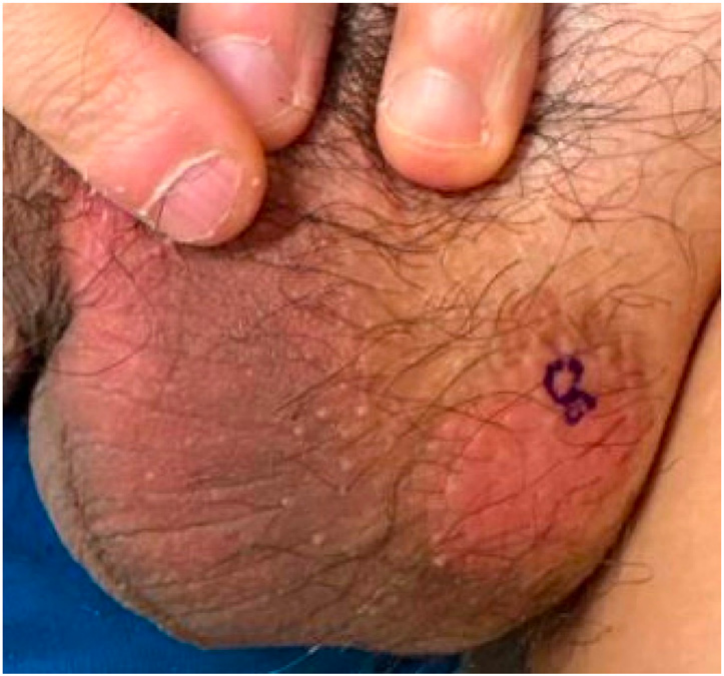
Pink plaque on the left side of the scrotum with peripheral hyperpigmentation.

**Fig 2:**
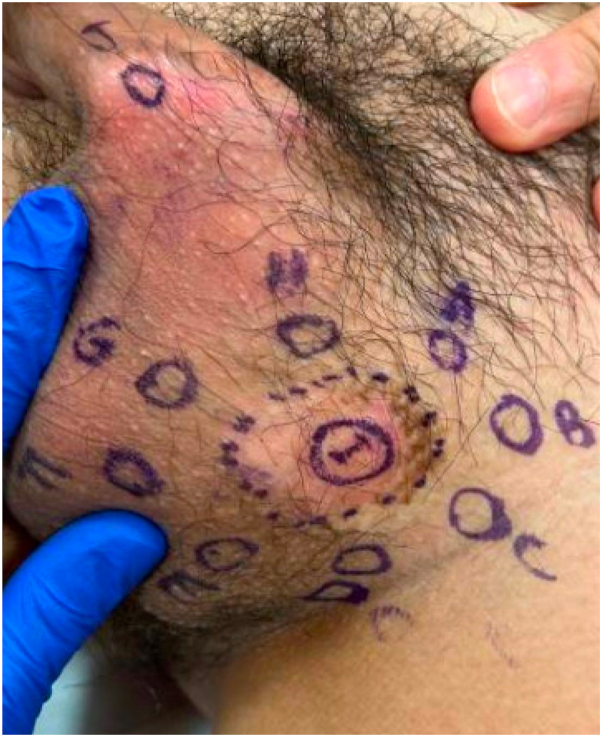
Map of scouting biopsies. Specimens D and I positive for extramammary Paget disease. All other specimens negative for malignancy.

An Asian male patient in his late 30s with an approximate 5-year history of HIV, currently on dolutegravir-lamivudine with undetectable viral load and CD4 count above 500, presented with pink plaques on the left side of the scrotum and left side of the penile base associated with mild pruritus and burning for at least 1 year (Fig 1). Two punch biopsies from the scrotum were obtained. Pathology showed clusters of epithelioid cells with ample myxoid cytoplasm within the epidermis. Neoplastic cells stained positive for CK7 and negative for CK20, suggestive of primary EMPD. Biopsy from the penile base was performed and was negative for EMPD. The patient was referred for MMS consultation and multidisciplinary cancer screening. During consultation for MMS, 10 additional scouting biopsies were obtained (Fig 2), including a repeat biopsy from a pink patch at the penile base (J). The central specimen (I) was positive for intraepidermal EMPD and the peripheral margin of specimen D was also positive. Remaining biopsies were negative for EMPD.

Because of immunosuppressed status, young age, and patient preference, extensive oncologic work up was performed to rule out secondary EMPD. Urine cytology was negative for high grade urothelial carcinoma. CA 19-9, CEA, PSA, and CA 15-3 were normal. IHC was negative for PD-L1 and progesterone receptor. Mutational burden testing was remarkable for ERBB3 mutation but otherwise showed no mutations requiring change in management or further testing. The patient was referred to urology and gastroenterology to rule out underlying malignancy. Renal ultrasound, scrotal ultrasound, cystoscopy, esophagogastroduodenoscopy, and colonoscopy were unrevealing.

**Fig 3:**
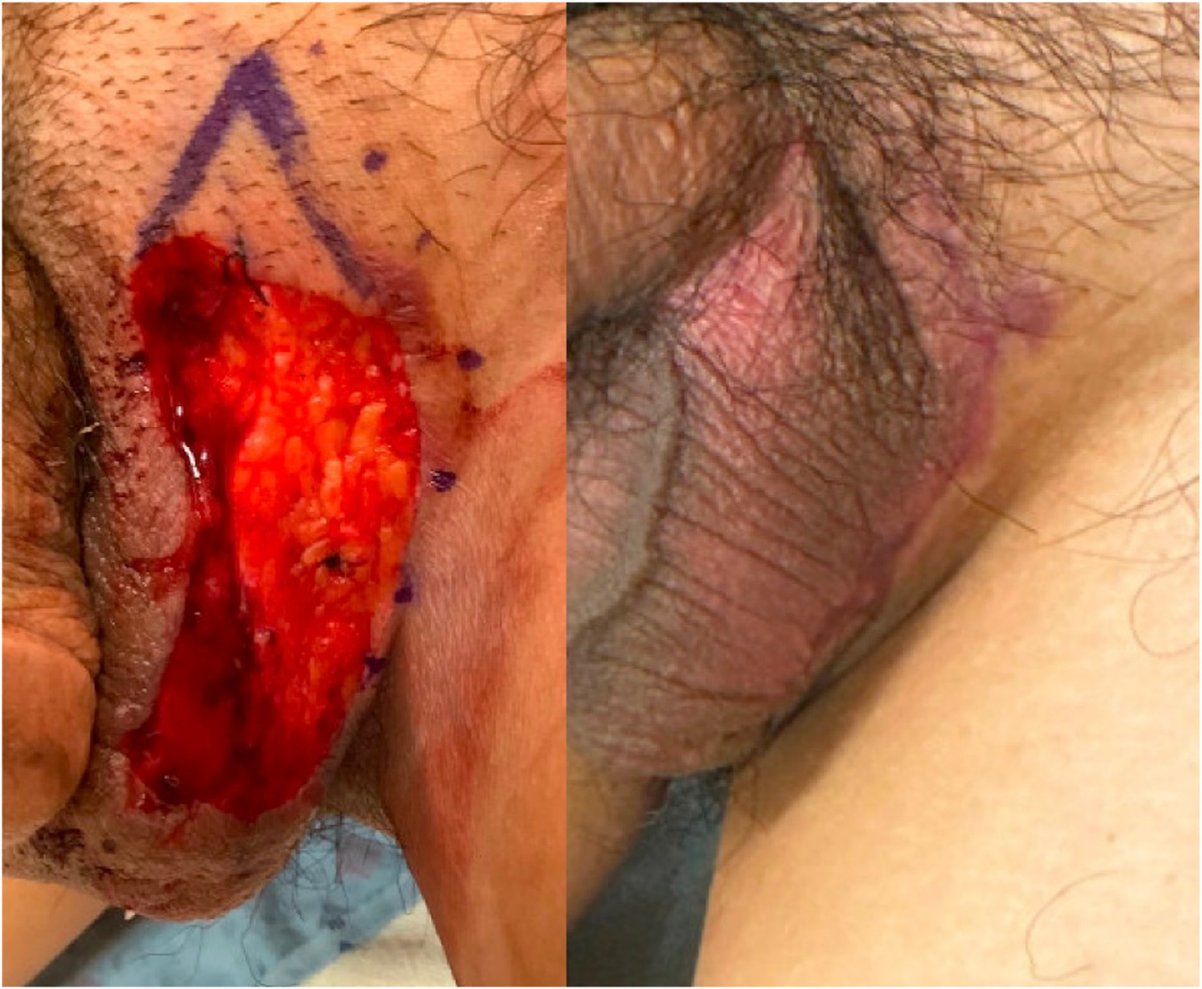
Mohs defect (*left panel*). Six weeks after Mohs micrographic surgery with intermediate primary repair (*right panel*).

The patient was treated with MMS with IHC analysis for CK7. Complete resection was obtained after 3 stages, and intermediate primary repair was performed the following day (Fig 3). The patient’s postoperative course was complicated by fever. Given the concern for possible surgical site infection, he was treated with a course of cefadroxil. He subsequently tested positive for COVID-19 and was advised to follow-up with his primary care provider for further evaluation and treatment.

## Discussion

This case report highlights a rare presentation of primary EMPD in a young, HIV-positive man. Paget disease does not demonstrate greater incidence in patients with HIV; however, severe immunodeficiency, as can be seen with AIDS, can be associated with unfavorable prognosis.^1^ To our knowledge, there has been 1 case report of EMPD in a 62-year-old, HIV-positive man in the literature. Similarly, the patient’s HIV infection was well-controlled and immunosuppression was not considered to be a contributing factor.^1^ EMPD has been reported in a 23-year-old man with immunosuppression secondary to adalimumab for hidradenitis suppurativa.^8^ No clear association between viral infections and EMPD has been established, and EMPD has been infrequently reported in the setting of concomitant condyloma acuminata and HIV.^1^^,^^4^^,^^7^

The diagnosis of EMPD is challenging owing to its rarity and clinical findings that may overlap with other entities such as eczema or intertrigo.^1^^,^^4^ Other differential diagnoses include leukoplakia, squamous cell carcinoma in situ, amelanotic superficial spreading melanoma, lichen sclerosus, inverse psoriasis, and vitiligo, especially for hypopigmented lesions.^1^^,^^6^ A high degree of suspicion is required when evaluating genital disease.

Treatment with wide local excision or MMS is often effective as subclinical disease and ill-defined borders are often present.^1^^,^^4^^,^^6^ One study reported a recurrence rate of 37.0% for wide local excision alone.^5^ However, recurrence rates ranging from 22% to 60% have also been reported for standard surgical treatments.^9^ Intraoperative IHC for CK7 during MMS has been shown to achieve the lowest local recurrence rates to date at 3.3% vs 25.9% for MMS without IHC.^9^ The patient described herein underwent MMS with IHC analysis for CK7 with complete resection and is being closely monitored. Although EMPD has rarely been reported in immunosuppressed and virally infected patients, whether these factors potentiate EMPD development has not yet been elucidated.

## Conflicts of interest

None disclosed.
